# Tsunami generation potential of a strike-slip fault tip in the westernmost Mediterranean

**DOI:** 10.1038/s41598-021-95729-6

**Published:** 2021-08-10

**Authors:** F. Estrada, J. M. González-Vida, J. A. Peláez, J. Galindo-Zaldívar, S. Ortega, J. Macías, J. T. Vázquez, G. Ercilla

**Affiliations:** 1 Institut de Ciències del Mar, CSIC., 08003 Barcelona, Spain; 2 Departamento de Matemática Aplicada, Escuela Politécnica Superior, Universidad de Málaga, 29071 Málaga, Spain; 3 Department of Physics, Universidad de Jaén, 23071 Jaén, Spain; 4 Departamento de Geodinámica, Universidad de Granada, 18071 Granada, Spain; 5 Instituto Andaluz de Ciencias de La Tierra (CSIC-UGR), Granada, Spain; 6 Departamento de Análisis Matemático, Facultad de Ciencias, Universidad de Málaga, Campus de Teatinos s/n, 29080 Málaga, Spain; 7 Instituto Español de Oceanografía, Centro Oceanográfico de Málaga, Puerto Pesquero s/n, 29640 Fuengirola, Spain

**Keywords:** Natural hazards, Ocean sciences

## Abstract

Tsunamis are triggered by sudden seafloor displacements, and usually originate from seismic activity at faults. Nevertheless, strike-slip faults are usually disregarded as major triggers, as they are thought to be capable of generating only moderate seafloor deformation; accordingly, the tsunamigenic potential of the vertical throw at the tips of strike-slip faults is not thought to be significant. We found the active dextral NW–SE Averroes Fault in the central Alboran Sea (westernmost Mediterranean) has a historical vertical throw of up to 5.4 m at its northwestern tip corresponding to an earthquake of Mw 7.0. We modelled the tsunamigenic potential of this seafloor deformation by Tsunami-HySEA software using the Coulomb 3.3 code. Waves propagating on two main branches reach highly populated sectors of the Iberian coast with maximum arrival heights of 6 m within 21 and 35 min, which is too quick for current early-warning systems to operate successfully. These findings suggest that the tsunamigenic potential of strike-slip faults is more important than previously thought, and should be taken into account for the re-evaluation of tsunami early-warning systems.

## Introduction

Tsunamis, catastrophic natural hazards that pose a significant threat to major infrastructure and many densely populated coastal regions, are generated by the rapid deformation of the seafloor due to fault or landslide activity^1–4^. Seismic strike-slip faults do not significantly displace the seafloor in flat-lying and smooth areas, and are therefore not generally considered as potential triggers of tsunamis^5–7^. Yet, tsunamis triggered by strike-slip faults have been reported worldwide as a result of either vertical seafloor displacements in over-steepened areas (e.g., the 1994 Mindoro earthquake^8^) and on restraining and releasing bends (the 1906 San Francisco earthquake^9,10^ and events in other areas of southern California^2^), or seismogenic submarine landslides (the 2010 Haiti earthquake^11^). The triggers of other historical strike-slip earthquake-related tsunamis, such as the 1999 Izmit tsunami^12^ and the 2012 and 2016 tsunamis in the Indian Ocean^13^, remain unknown. To date, despite the noteworthy vertical offsets at the tips of strike-slip faults, these faults have not been considered a main tsunamigenic source.

**Figure 1:**
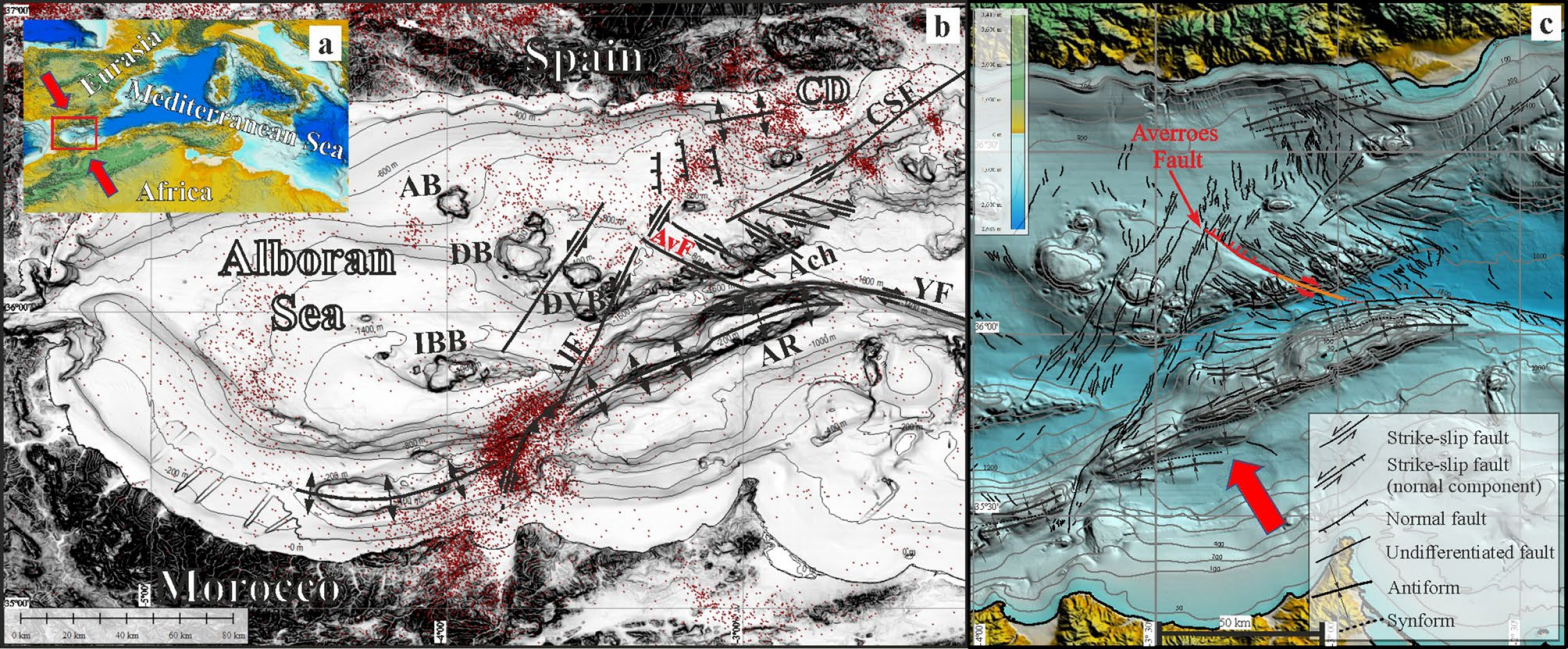
The Averroes Fault in the tectonic framework of the Alboran Sea. (**a**) Location of the study area; (**b**) Multibeam bathymetric map displaying the main NNE-SSW shear zone seismicity (red dots) and main tectonic features and seamounts; (**c**) detailed tectonic map of the central Alboran Sea highlighting the presence of the Averroes Fault northern segment in red and southern segment in orange. Modified from Estrada et al.^15^. *ACh* Alboran Channel, *AR* Alboran Ridge, *AB* Algarrobo Bank, *DB* Djibouti Bank, *DVB* Djibouti Ville Bank, *IBB* Ibn Batouta Bank, *CD* Campo de Dalias, *YF* Yusuf Fault, *AIF* Al Idrisi Fault, *AvF* Averroes Fault, *CSF* Carboneras-Serrata Fault. Red arrow indicates direction of tectonic indentation. (Figures generated using Globalmapper v.19, https://www.bluemarblegeo.com, and mounted with CorelDRAW v. X7, https://www.corel.com).

The strike-slip Averroes Fault is located in the Alboran Sea (westernmost Mediterranean Sea), which has been a tectonically active basin since the late Miocene^14^ (Fig. 1). The Alboran Sea is deformed by strike-slip faults under laterally unlocked tectonic indentation driven by Eurasian-African plate convergence^15^ at a rate of 4.5 mm/yr^16^ (Fig. 1) The Alboran Sea, whose Iberian coast annually receives the highest number of tourists from all of Europe, has been historically afflicted by tsunamis^17–19^. Historical records show the simultaneous occurrence of tsunamis striking the Adra and Malaga coasts at 365 CE^17^. However, little is known about the tsunamigenic potential of the faults in this basin.

**Figure 2:**
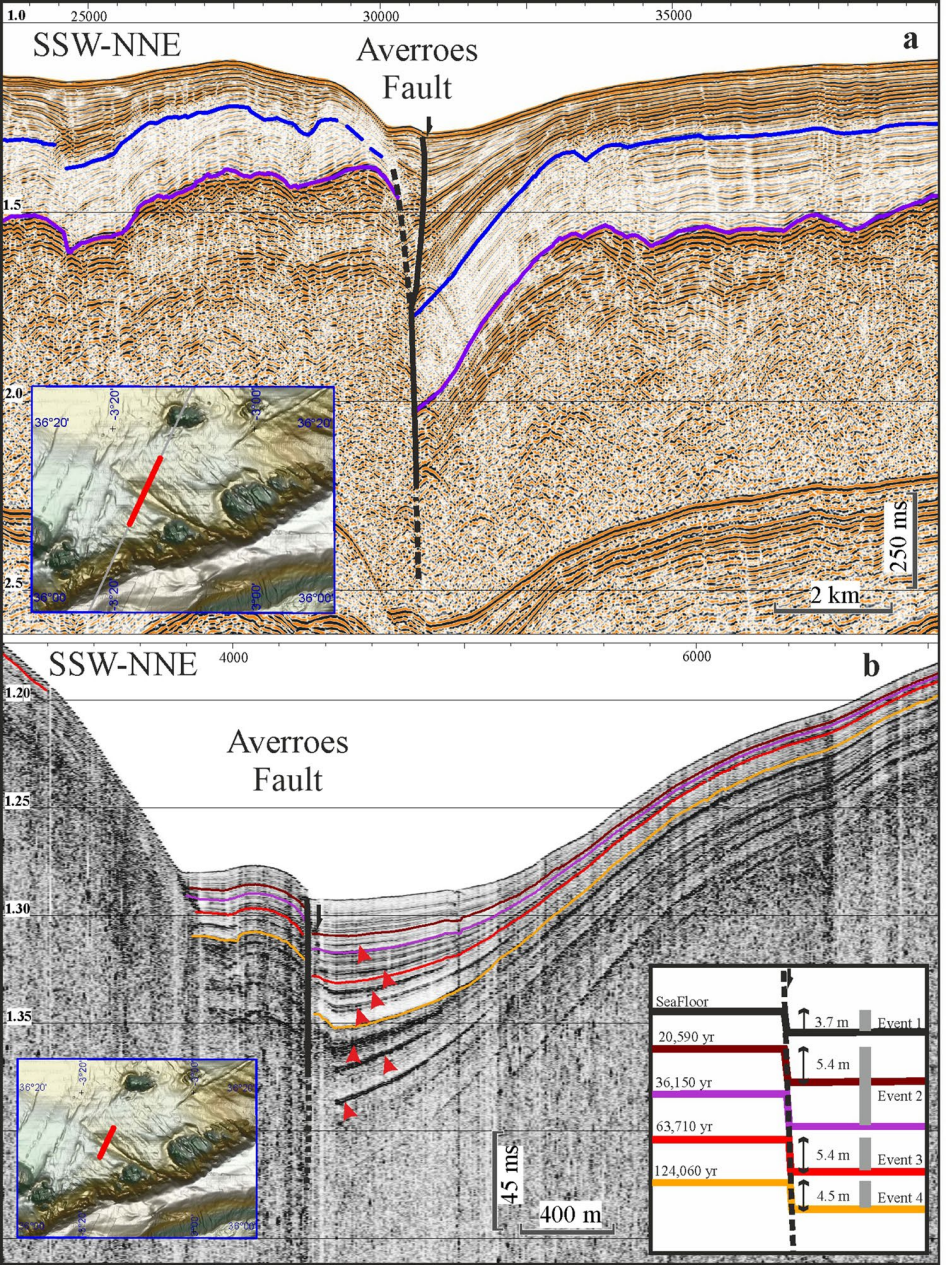
Seismic profiles illustrating the Averroes Fault. (**a**) Airgun seismic profile showing the deep structure. Legend: purple line corresponds with top of Miocene and blue line with base of Quaternary. Horizontal and vertical scale respectively in metres and seconds, two-way travel time (twtt). Vertical exaggeration × 8.7; (**b**) Parametric TOPAS profile (ultra-high-resolution) illustrating the upper reach (75 m) of the Averroes Fault. Inlet shows chronostratigraphic boundaries and fault events over the last 124,060 years. Red arrows indicate co-seismic wedges. Horizontal scale in metres and vertical scale in seconds (twtt). Vertical exaggeration × 12.4. (Figures generated using IHS Kingdom v. 2017, https://ihsmarkit.com, and mounted with CorelDRAW v. X7, https://www.corel.com).

To resolve this problem, we identified the NW–SE dextral Averroes Fault as the structure in the fault system with the strongest evidence of recent and active seafloor offset at its northern termination (Figs. 1b,c and 2). Subsequent modelling the tsunamigenic potential of this structure reveals the potential for tsunami generation triggered by vertical offset at the tip of a strike-slip fault, providing knowledge crucial for reviewing potential tsunami hazards related to strike-slip faults worldwide.

## Results

### The strike-slip Averroes fault

The Averroes Fault is a component of the NW–SE conjugate dextral strike-slip fault set of the main NNE-SSW shear zone crossing the Alboran Sea^15^ (Fig. 1b). The Averroes Fault, which is predominantly affected by shallow earthquakes at present (up to 15 km deep^20^), has a steeply dipping main fault surface with a length of 38 km (Figs. 1b,c and 2) that splits upward into two faults surfaces, the eastern one being currently deforming the seafloor (Fig. 2a).

The main Averroes Fault comprises two main segments: the southern segment (16 km long) horizontally displaces the seafloor surface by 4.1 km with dextral kinematics, while its northern segment (22 km long) has a maximum vertical offset of 470 m at its tip (Fig. 2a), with a northeastern downthrown block that creates a half-graben-like feature (Figs. 1c and 2). Tectonic activity initiated along the Averroes Fault during the late early Pliocene^21–24^, giving it an age of 4.57 Ma^21,25^. Considering the age of the fault and the vertical offset along its northern segment, we calculate an average vertical slip rate of 0.1 mm/yr.

Activity of the Averroes Fault is driven by the tectonic inversion of the central Alboran basin^15^. This activity has a co-seismic character evidenced in our ultra-high-resolution parametric seismic profiles by interbedded sedimentary wedges (up to 4.5 to 5.4 m thick) vertically stacked which represent mass-wasting events coming from the upthrown block (Fig. 2b). We have established a chronostratigraphic control of activity spanning the last 124,000 yr (Fig. 2b). From young to old, four events can be seen, with fault throws of 3.7 (seafloor), 5.4, 5.4 and 4.5 m (unloaded successive fault offsets), and with ages of recent, 20,590 yr, 63,350 yr and 124,060 yr, respectively (Fig. 2b and Suppl. Figure 1). We therefore propose an approximate average recurrence period of approximately 31,000 yr.

### Seafloor deformation and tsunamigenic potential

Next, we modelled the tsunamigenic potential of the vertical seafloor offsets related to the fault tip of the NW segment of the Averroes strike-slip Fault. Although seafloor deformation may also occur by horizontal displacement of slope areas in the southern segment, it would be reasonable to assume that horizontal displacement away from the tip might not lead to significant vertical offset.

**Figure 3:**
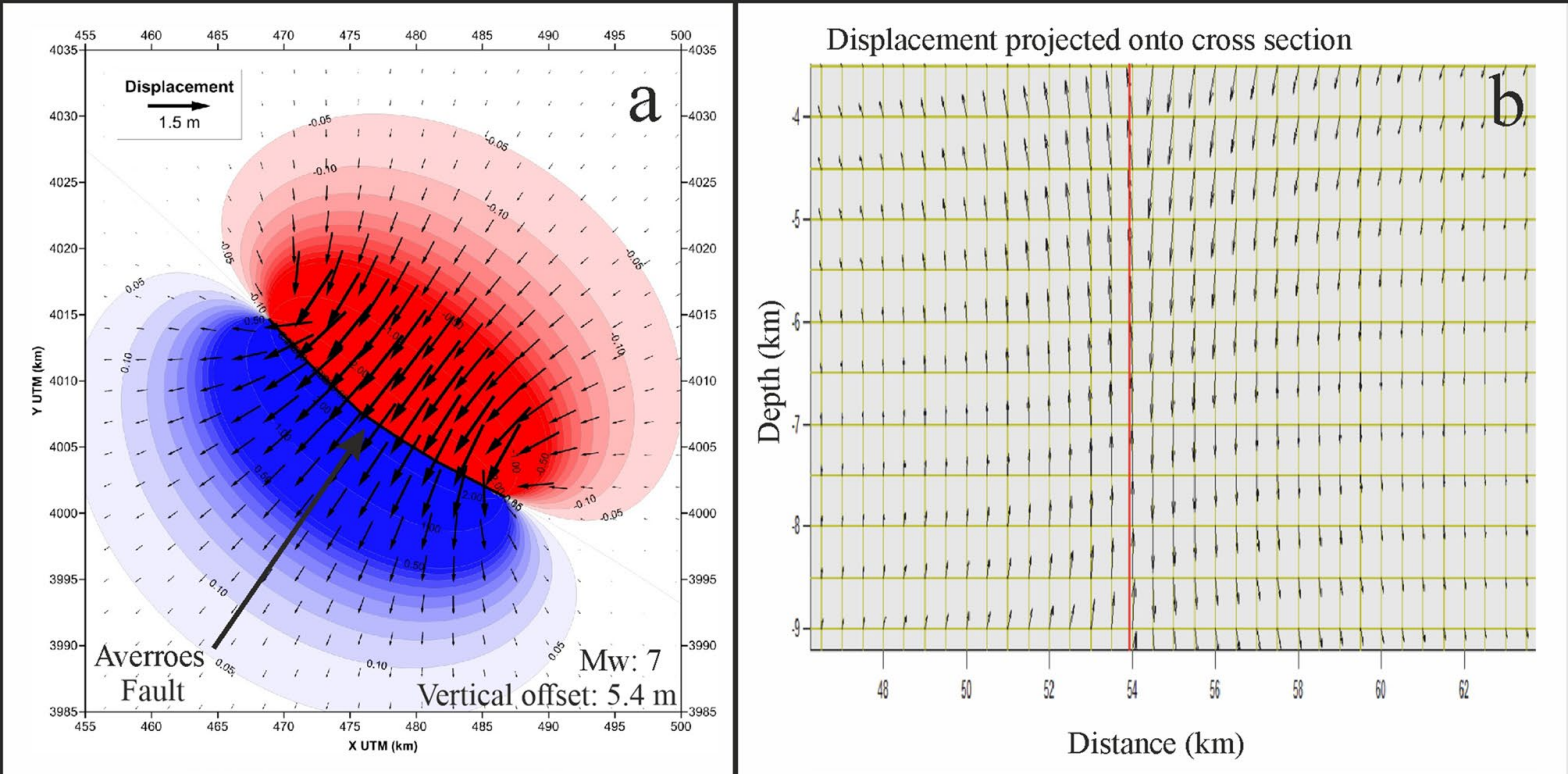
Seafloor deformation. Computed deformation pattern of the crust affected by the Averroes Fault for a vertical offset of 5.4 m and an associated magnitude of Mw 7. (**a**) Seafloor plan view: red indicates downthrow and blue indicates uplift; (**b**) Vertical section: red line represents the Averroes Fault trace. (Figures generated with Coulomb, v. 3.3, USGS, https://www.usgs.gov/software/coulomb-3 and Surfer, v. 12.0.626, Golden Software, https://www.goldensoftware.com and mounted with CorelDRAW v. X7, https://www.corel.com).

We employed Okada’s approach^26,27^ to calculate deformation, by determining a displacement on the fault plane of the SW (uplifted) block equal to one-half of the net slip; i.e., the displacement of the SW block was equivalent in magnitude to the displacement of the NE (downthrown) block. We modelled a rapid co-seismic displacement of the seafloor considering a vertical fault with a length of 22 km extending to a depth of 10 km and a uniform net slip of 5.4 m (with the NE block downthrown). This net slip corresponds to the historical maximum throw, and was determined for two of the four events mentioned above. Our model suggests a corresponding seismic moment equal to 3.88 × 10^26^ dyn/cm and an earthquake magnitude of Mw 7. Both the vertical and the horizontal computed deformations are depicted in Fig. 3, which demonstrates that deformation lobes affect both fault blocks with vertical displacements of 0.1 m even at distances (perpendicular to the fault plane) of 17 km for the simulated Mw 7.0 event (Fig. 3). The maximum horizontal displacements perpendicular to the fault plane are on the order of 1.7 m.

**Figure 4:**
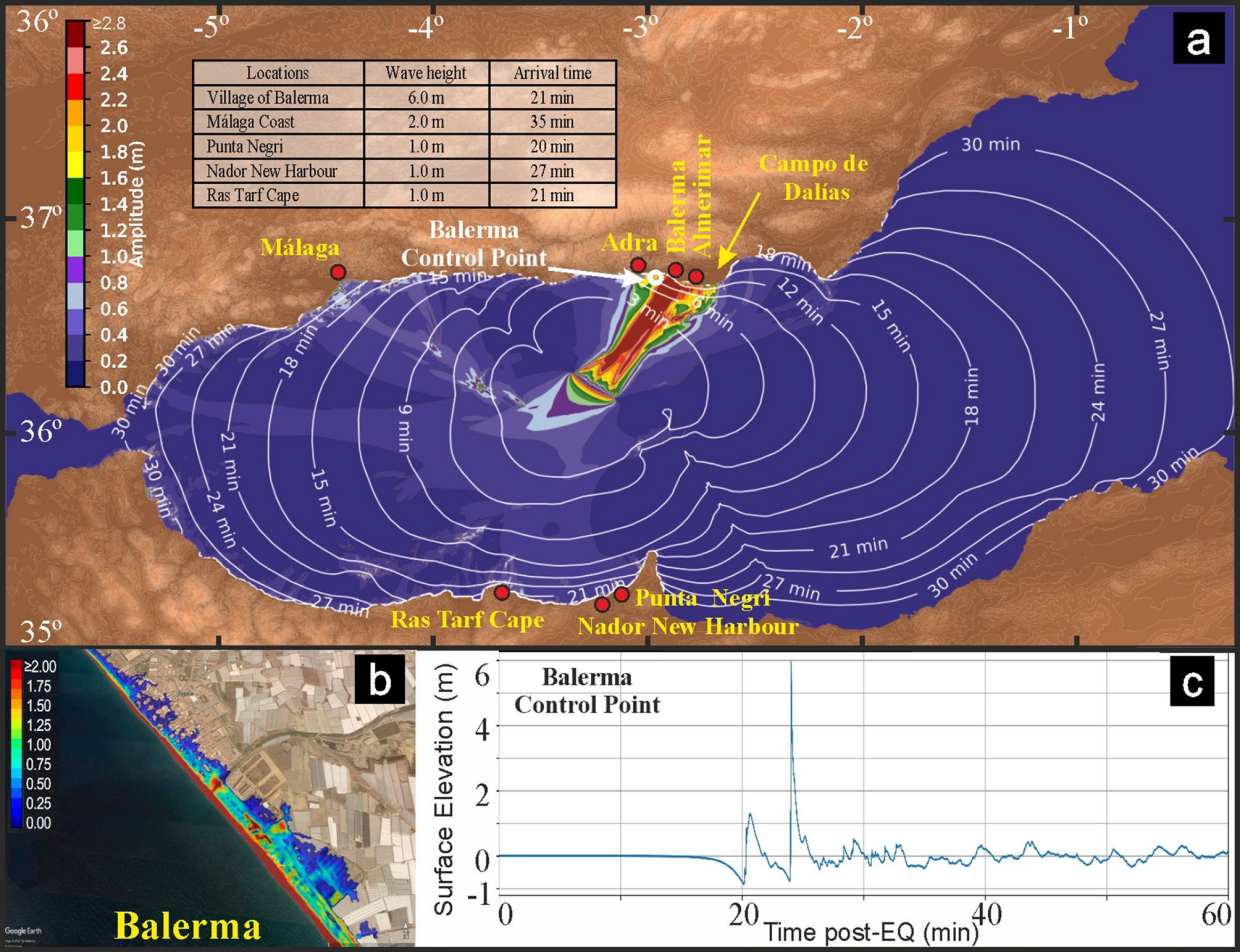
Modelled tsunami for the Averroes Fault. (**a**) Tsunami propagation considering a vertical offset of 5.4 m and an associated magnitude of Mw 7. Colour scale represents wave heights in metres and contours represent first arrival times in minutes. The highest wave heights and their arrival times impacting coastal localities are shown in the inset table. (**b**) Balerma tsunami water-level inundation. Colour scale in meters. (**c**) Tsunami wave height at Balerma control point based on seasurface time series simulated by Tsunamni-HySEA. (Figures generated using Python, v. 3.7.3, https://www.python.org, and Matplotlib v. 3.4.2, https://matplotlib.org and mounted with CorelDRAW v. X7, https://www.corel.com).

Next, we modelled the tsunami generation that might occur as a result of a vertical throw of 5.4 m on the northern segment of the Averroes Fault, using the non-linear hydrostatic shallow-water model Tsunami-HySEA applied to a high-resolution ambient grid (~ 50 m). Despite the hydrostatic nature of the numerical model used, it have been shown^28^ that the hydrostatic version of the Tsunami-HySEA model is capable of accurately assess tsunami hazard as runup estimations agreed with lab data and essentially coincide with non-dispersive simulation results. We identified a heterogeneous tsunami propagation pattern, comprising two branches orthogonal to the fault trace; the main branch directed to the NE and the minor one directed to the SW (Fig. 4a and Suppl. Video 1). The NE branch has a straight path, and reaches land in the area of Campo de Dalías (Fig. 4a, Suppl. Figure 2 and Suppl. Videos 2 and Video 3); a positive wave with maximum height (6 m) and shortest arrival time (21 min) occurs near the village of Balerma (Fig. 4a,c and Suppl. Video 2). In contrast, the SW branch initially corresponds to deep waters, and the propagating tsunami impinges against three elongated seamounts acting as morphological barriers, namely, the relatively long ENE-WSW Alboran Ridge, the E-W Ibn Batouta and three seamounts aligned NW–SE (Algarrobo, Djibouti and Djibouti Ville banks) (Figs. 1a, 4a and Suppl. Video 1). This impingement modifies the tsunami propagation path, forcing it to split into three subbranches directed to the NW and approximately to the W and S. The former reaches the Málaga coast with a wave height of up to 2 m and an arrival time of up to 35 min (Fig. 4a, Suppl. Figure 2 and Suppl. Video 4), whereas the latter reaches landfall along the Moroccan coast, in locations such as Ras Tarf cape (wave height 1 m; arrival time 21 min), Punta Negri (wave height 1 m; arrival time 20 min) and the new Nador Harbour (Port Nador West Med) (wave height 1 m; arrival time 27 min) (Fig. 4a, Suppl. Figure 2 and Suppl. Video 1).

## Discussion

After an earthquake strikes, it is crucial for early-warning systems to issue a rapid assessment while minimizing false alerts, based on the event magnitude, focal mechanism and potential tsunamigenic trigger.

The Averroes Fault in the Alboran Sea (Fig. 1a,b) with offsets that can reach 5.4 m (Fig. 2), suggesting earthquake magnitudes up to Mw 7, provides a fundamental opportunity to analyze this tsunamigenic mechanism, namely, the vertical offset of the seafloor concentrated at the northern fault tip.

Our modelling of tsunami propagation related to seafloor deformation along the Averroes Fault (Fig. 4 and Suppl. Video 1) provides a means to assess the potential tsunamigenic hazard of strike-slip fault tips. As already mentioned, despite the limitation in the tsunami model used, not able to reproduce the dispersive characteristics of the generated waves, it have been shown^28^ that regarding hazard assessment, the model used provides accurate estimations for runup as main indicator of the hazard. The propagation of tsunami is controlled by fault kinematics^5^ and we found that the vertical throw at the tip of the Averroes Fault determines the onset of positive and negative waves. The vertical deformation lobe of the NE downthrown block triggers an initial negative wave followed by a striking positive wave raising up to 6 m above the usual water level at Balerma coast (Fig. 4 and Suppl. Video 2). Contrasting, the vertical deformation of the SW uplifted block forms an initial positive wave with a remarkable negative wave dropping up to 1.5 m in Malaga and 1.5 m in Moroccan coasts, and wave trains with significant withdrawal in both areas (Suppl. Figure 2).

The propagation of tsunami is also controlled by the interplay between fault orientation and seafloor morphology^5,6^, and we found that the NW–SE-oriented Averroes Fault focuses waves mainly towards the NE and, to a lesser extent, the SW. The NE branch is predicted to cross an area of smooth bathymetry, enabling linear propagation towards the Campo de Dalías coast, which is characterized by tourist beaches and dominated inland by greenhouse agriculture (Fig. 4 and Suppl. Video 1); likewise, the coastal proximity of the villages of Balerma, Almerimar and Adra, and the short propagation time increase their vulnerability to tsunamis (Fig. 4, Suppl. Figure 2 and Suppl. Videos 2 and Video 3). The NW subbranch focuses the highest waves towards the Malaga coast, which exhibits a moderate tsunami hazard that may affect infrastructures and beaches (Fig. 4, Suppl. Figure 2 and Suppl. Videos 1 and Video 4). Moreover, the coastline in this region becomes densely populated in summer, with large stretches of beach that can be fully occupied. Meanwhile, the S subbranch influences the Moroccan coast, but to a lesser extent (Fig. 4, Suppl. Figure 2 and Suppl. Video 1).

Our findings demonstrate the need to reevaluate tsunamigenic hazards related to strike-slip fault tips. Such faults are found predominantly in marine areas related to transform plate boundaries, such as the northern San Andreas Fault^29^ and the northeastern Caribbean plate^30^, as well as small seas, such as the Marmara Sea^31^. Likewise, vertical offsets at fault tips may be found along strike-slip faults related to segmented subduction zones, such as the Nazca plate below the Ecuador-Colombia segment of the South American plate margin^32^. All the mentioned examples, are similar enough to the Averroes Fault and might also pose a significant threat to the local population.

Here, we demonstrate that following a seismic event, vertical offset at the NW tip of the Averroes Fault has the potential to generate destructive tsunamis in the westernmost Mediterranean, with rapid arrival times at densely populated coastlines (21 min) that are too short for existing early-warning systems to operate properly. Moreover, this study highlights the need for coastal communities worldwide to review the tsunamigenic potential of strike-slip faults through vertical offsets near their tips as a new tsunamigenic mechanism that may be of great importance along transform and segmented convergent plate boundaries characterized by submarine strike-slip faults. These findings justify the necessity of considering the tsunamigenic hazard potential of strike-slip faults to improve the accuracy of tsunami early-warning systems in geodynamic contexts of tectonic indentation, transform plate boundaries and subduction zones.

## Methods

### Marine geophysics

The geologic history and structure of the Averroes Fault were studied by means of seismic profiles and swath bathymetric data. The seismic profiles come from the following 17 cruises: AS, CONOCO Cab-01, R/V Robert D. Conrad cruise, DBS, EAS, Fauces, Fauces-1bis, GC-83-2, GC-90-1, GC-90-2, He-91-3, RRS Charles Darwin cruise, Marsibal, Montera, RAY, SAGAS, (http://gma.icm.csic.es/sites/default/files/geowebs/OLsurveys/index.htm). The cruises were independent to this study, except 4 of them (Montera, GC-90–1, SAGAS, Fauces-1) where the surveys of the Averroes Fault was included in the cruise objectives and provided new and relevant information; these 4 cruises were led by scientists of the Continental Margins Group from the Institute of Marine Science, ICM-CSIC, and were conducted onboard the Spanish research vessels García del CID, Hespérides; Sarmiento de Gamboa and Angeles Alvariño. The seismic profiles have different resolutions (high and ultrahigh) and utilize different techniques: multi-channel seismic (MCS), single channel and parametric. Multi-channel seismic profiles were downloaded from the Spanish Hydrocarbon Technical Archive (https://geoportal.minetur.gob.es/ATHv2/welcome.do) and they are commercial data from oil companies. These profiles have a vertical standard resolution of tens of meters with a penetration of up to 10 s. The single-channel profiles were obtained with airgun systems (140 to 530 c.i.), have an average vertical resolution of < 15 m of few meters time with a penetration of up to < 3 s seconds. The parametric seismic profiles were acquired with the TOPAS (TOpographic Parametric Echosounder) whose vertical resolution is about < 30 ± 40 cm within the upper 150 ms of the sediment column. The seismic lines were integrated into a IHS Kingdom project for their correlation and interpretation.

The swath bathymetric data were recorded with a SIMRAD EM120 multibeam echosounder with a frequency of 12 kHz. Multi-beam bathymetry datasets independent to this study were also compiled and integrated for the present study. These bathymetries were obtained from the MARSIBAL and Fauces 1bis projects and the Fishing General Secretary (Spanish Government). The data are available at a repository (http://gma.icm.csic.es/sites/default/files/geowebs/OLsurveys/index.htm). The Global Mapper software, a versatile GIS application from Blue Marble Geographics, was selected for integrating all those bathymetries in order to generate a gridded bathymetric map at 50 m. The vertical resolution was approximately 0.025% of the water depth. The mapping of the Averroes Fault trace and its analysis on the multibeam map was also done with drawing and measurement tools in Global Mapper and IHS Kingdom project.

For plotting the epicentre locations at the seafloor of the Alboran Sea, the seismicity database of the Spanish National Geographic Institute (IGN) (www.ign.es) was used. The epicentre datasets were plotted on the multibeam bathymetric map using visualization tools from the Global Mapper.

### Chronostratigraphy

The available scientific well information for the study area (ODP Site 977) was integrated into the IHS Kingdom project with seismic lines for their chronostratigraphy correlation and interpretation. A precise chronology of the seismic stratigraphic boundaries was developed through an age calibration based primarily on data from that Site (Suppl. Figure 1). In order to confirm the chronology, the chronostratigraphic boundaries were also correlated with those in commercial and scientific wells for across the entire Alboran Basin^33^. The velocity-to-depth (ms to m) conversion was performed using the speed of sound (1500 m/s) for the parametric profiles (Suppl. Figure 1) and a weighted average velocity (1779 m/s) from ODP Site 976^34^ for the multi- and single-channel profiles.

### The Averroes fault rate of tectonic activity

The average vertical slip rate (0.1 mm/yr) results from divide the vertical offset (470 m) by the age of the oldest materials affected by the Averroes Fault (4.57 myr). In the same way, the averaged period of fault recurrence (31,000 yr) has been calculated dividing the oldest known age (124,060 yr) by the number of fault events (4).

### Seafloor deformation

The crustal deformation at the seafloor generated by a given earthquake along the Averroes Fault was computed using the Coulomb 3.3 code^35,36^, in which calculations were performed using an established approach^26,27^ assuming an elastic half-space with uniform elastic properties. The fault was modelled using different vertical planes to better fit the curved geometry of the fault trace. All of these planes extended from the surface of the seafloor to a depth of 10 km, where the majority of hypocentres near the Averroes Fault are located. Typical values of 0.25 for Poisson’s ratio and 8 × 10^25^ bar for Young’s modulus were applied.

### Tsunami model

Tsunami-Hyperbolic Systems and Efficient Algorithms (Tsunami-HySEA)^37,38^ is a finite-volume numerical hydrostatic model that solves the 2D non-linear shallow water equations in spherical coordinates. It has been developed by the EDANYA group of the University of Malaga specifically for simulations of seismically induced tsunamis. This model, based on a graphic processing unit (GPU) architecture, is robust, reliable and accurate. The combination of this kind of numerical model with an efficient GPU results in a faster than real-time (FTRT) numerical model capable of simulating the generation, propagation and inundation of a tsunami in a region covered by a grid with several million cells in only a few minutes. This model has been extensively validated under the standard benchmarks proposed by the National Tsunami Hazards Mitigation Program (NTHMP) of the U.S.A.^28,39^ and has been extensively tested in several scenarios and compared with other well-established tsunami models^40,41^.

Tsunami-HySEA has been implemented using CUDA and MPI in order to take advantage of the massive parallel architecture of multi-GPU clusters, so that the computing time required could be dramatically reduced with respect to the use of a single CPU core or even a multi-core processor and, at the same time, numerical resolution could be increased still computing extremely fast. The Tsunami-HySEA model includes many features such as various options for the initial condition (as the computation of the initial seafloor deformation using Okada^27^ (1992) model or support for rectangular or triangular faults among others), it implements two-way nested meshes, direct output of time series from a list of points of interest, etc^42^. A 2D domain decomposition is performed, and load balancing techniques are also used considering the wet and dry zones and the nested meshes, so that the computational load of all the MPI processes is as similar as possible. The entire numerical computation is carried out in multi-GPUs, using double numerical precision, including the nested meshes processing. Multiple CUDA kernels have been implemented, and CUDA streams are used to compute in parallel different meshes in a same level of the grid hierarchy. Furthermore, the MPI communications can overlap with kernel computations and memory transfers between CPU and GPU memory in order to increase the efficiency of the solver. By means of this very efficient implementation, the model is able to simulate 8 h of real time tsunami in the Mediterranean Sea (in a mesh with 10 million volumes and a resolution of 30 arc-sec) in 257 s using two NVIDIA Tesla P100, or even in 284 s with one NVIDIA Tesla V100.

Bathymetric DEM data has been extracted from the 15 arc-sec resolucion global GEBCO database. Topographic data has been extracted from the MDT05 DEM with 5 m resolution provided by the IGN (National Geographic Institute from Spain). The topobathymetric ambient grid covers the Alboran Sea from 5.0° W to 1.8353° W and 35.0598° N to 36.8499° N (Fig. 4) with a resolution of 1.611 arc-sec (~ 50 m). The ambient grid contains 28.284 million cells. Six high-resolution nested grids with a resolution of 0.201 arc-sec 315 (~ 6 m) and 52.522 million cells, has been defined along the Spanish coasts where the impact of 316 the tsunami is more important (Fig. S3). Mean sea level is used as initial condition as tides are negligible in this area of the Mediterranean. Friction Manning coefficient is set to 0.02 and CFL stability number is set to 0.5.

## Supplementary Information

Supplementary Information.

Supplementary Video 1.

Supplementary Video 2.

Supplementary Video 3.

Supplementary Video 4.
