# Supplementary material for: Tsunami generation potential of a strike-slip fault tip in the westernmost Mediterranean
Source: Sci Rep. 2021 Aug 10;11:16253. doi: 10.1038/s41598-021-95729-6 (PMC8355334; doi:10.1038/s41598-021-95729-6)
Supplement: Supplementary file 1 — Supplementary Information. [file 41598_2021_95729_MOESM1_ESM.docx]

**SUPPLEMENTARY MATERIAL**

**Tsunami generation potential of a strike-slip fault tip in the westernmost Mediterranean**

*F. Estrada, J. M. González-Vida, J. A. Peláez, J. Galindo-Zaldívar, S. Ortega, J. Macías, J. T. Vázquez, G. Ercilla


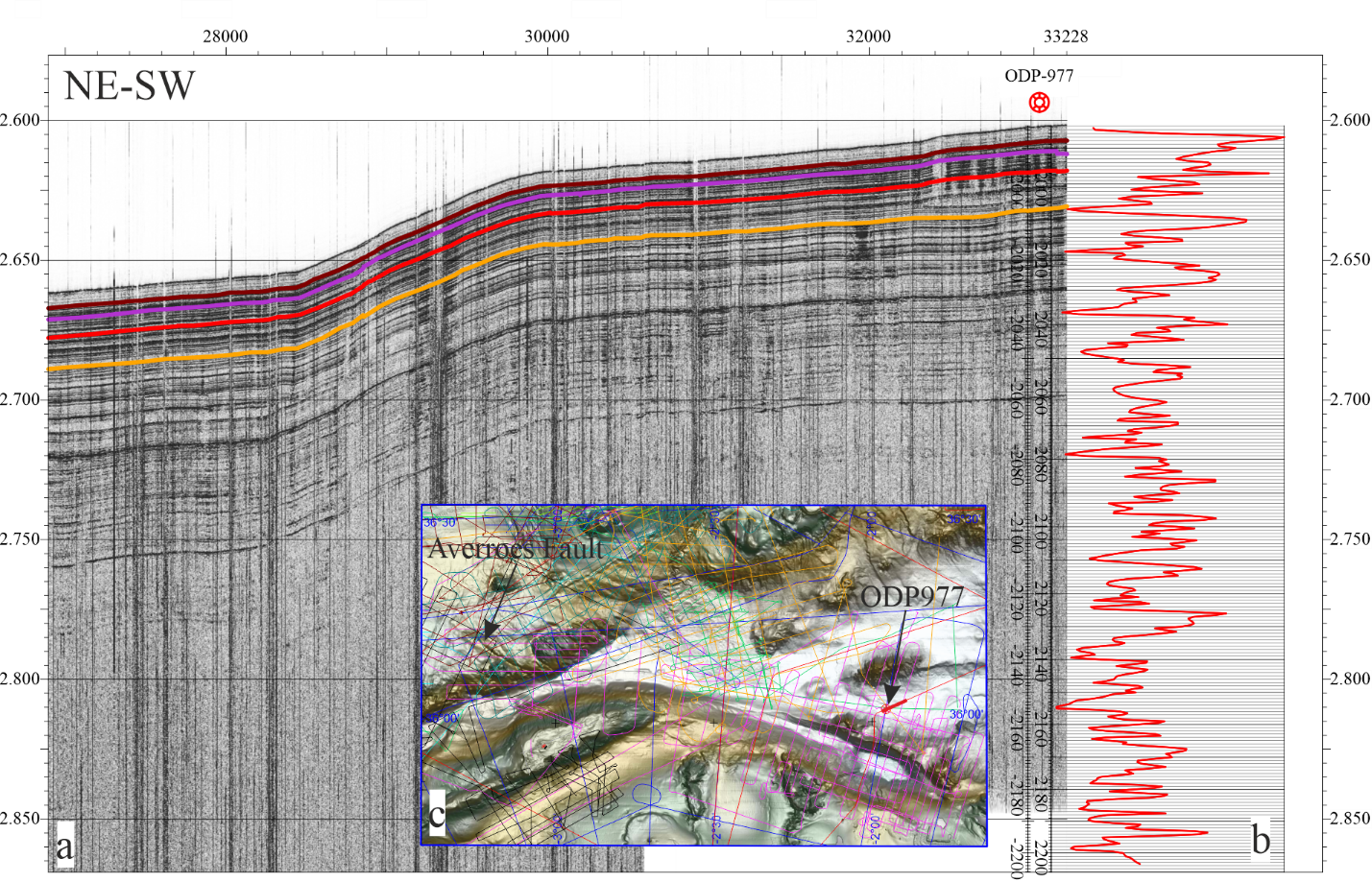


Supplementary Fig. S1: **Chronostratigraphy correlation.** Correlation between the TOPAS parametric seismic profile (ultra-high-resolution**)** (a) and oxygen-18 isotope curve; (b) stablished by Martrat^1^ et al. (2004) at ODP Site 977 well through a dense net of parametric seismic profiles. Seismic profile and well locations in c; there, also the seismic profiles (coloured lines) crossing the Averroes Fault that have been used for the strike-slip characterization can been seen. Horizontal and vertical scales respectively in meters and seconds (two-way travel time). (Figures generated using IHS Kingdom v. 2017, https://ihsmarkit.com, and mounted with CorelDRAW v. X7, https://www.corel.com).


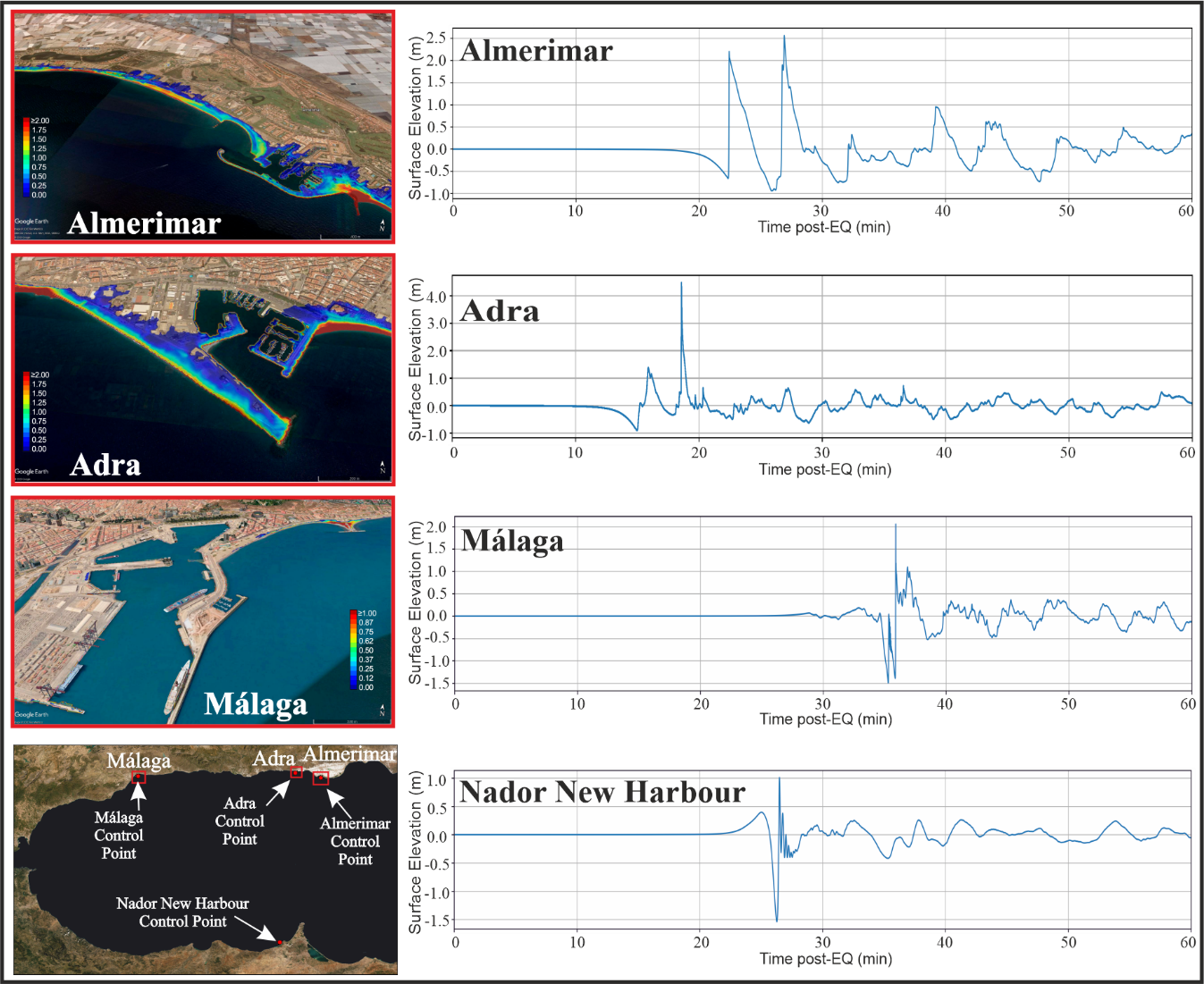


Supplementary Fig. S2: **Tsunami impact**. Satellite images from Google Earth (red squares) showing superimposed the tsunami water-level inundations of the Almerimar, Adra and Málaga zones; colour scale in metres. Plots represent tsunami wave height at control points based on seasurface time series simulated by Tsunami-HySEA stations (red dots). (Figures generated using Python, v. 3.7.3, https://www.python.org, and Matplotlib v. 3.4.2, <https://matplotlib.org> and mounted with CorelDRAW v. X7, https://www.corel.com).


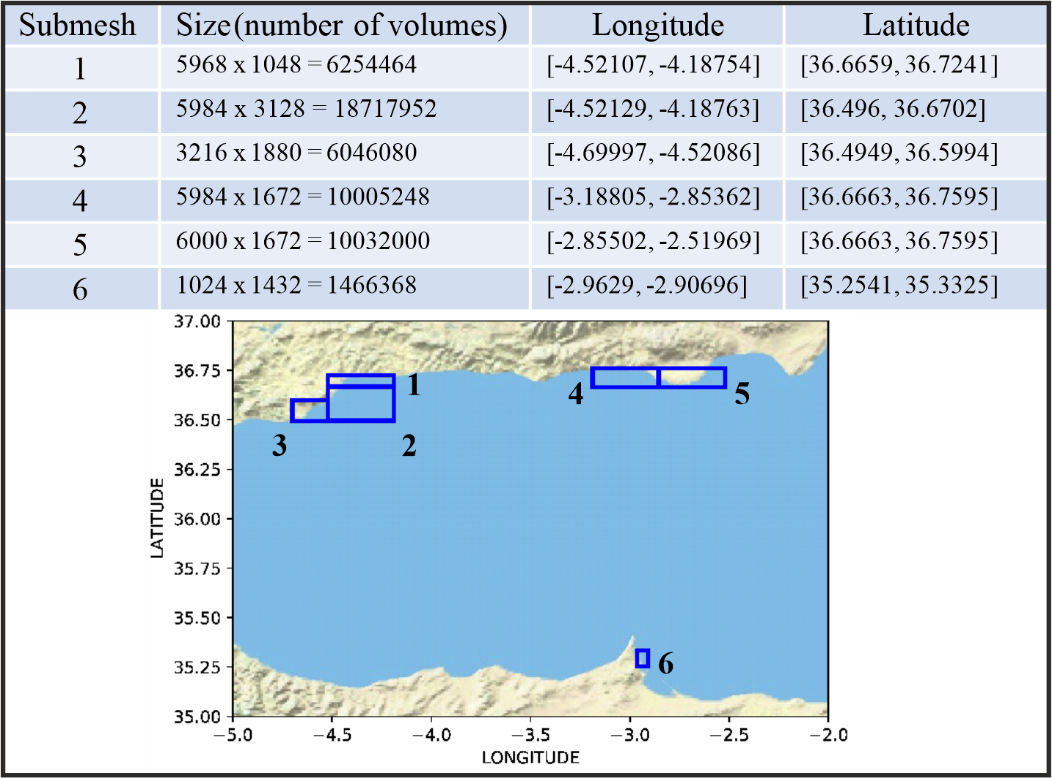


Supplementary Figure S3: **Nested meshes**. Table showing the size and location of the 6 nested meshes. The figure below displays their situation in the Alboran Sea. (Figure generated using Python, v. 3.7.3, https://www.python.org, and Matplotlib v. 3.4.2, <https://matplotlib.org> and mounted with CorelDRAW v. X7, https://www.corel.com).

Supplementary Video 1: tsunami propagation video of the Alboran Sea. Video length 31 seconds for 47 minutes of tsunami propagation.

Supplementary Video 2: tsunami propagation video of the Balerma zone Video length 1 minute for 1 hour of tsunami propagation.

Supplementary Video 3: tsunami propagation video of the Almerimar zone. Video length 1 minute for 1 hour of tsunami propagation.

Supplementary Video 4: tsunami propagation video of the Málaga zone. Video length 50 seconds for 1 hour of tsunami propagation.

References

1. Martrat, B., Grimalt, J. O., Lopez-Martínez, C., Cacho, I., Sierro, F. J., Flores J.A. et al. Abrupt temperature changes in the Western Mediterranean over the past 250,000 years. *Science*, **306** (5702), 1762-1765. <https://doi.org/10.1126/science.1101706> (2004).
